# Image-based 3D canopy reconstruction to determine potential productivity in complex multi-species crop systems

**DOI:** 10.1093/aob/mcw242

**Published:** 2017-01-08

**Authors:** Alexandra J. Burgess, Renata Retkute, Michael P. Pound, Sean Mayes, Erik H. Murchie

**Affiliations:** 1 Division of Plant and Crop Sciences, School of Biosciences, University of Nottingham, Sutton Bonington LE12 5RD, UK; 2 Crops For the Future, Jalan Broga, 43500 Semenyih Selangor Darul Ehsan, Malaysia; 3 School of Life Sciences, Gibbet Hill Campus, The University of Warwick, Coventry CV4 7AL, UK; 4 School of Computer Science, University of Nottingham, Jubilee Campus, Wollaton Road, Nottingham NG8 1BB, UK

**Keywords:** 3D reconstruction, Bambara groundnut (*Vigna subterranea* (L.) Verdc.), canopy architecture, canopy productivity, intercropping, light interception, photosynthesis, proso millet (*Panicum miliaceum*), ray tracing

## Abstract

**Background and Aims** Intercropping systems contain two or more species simultaneously in close proximity. Due to contrasting features of the component crops, quantification of the light environment and photosynthetic productivity is extremely difficult. However it is an essential component of productivity. Here, a low-tech but high-resolution method is presented that can be applied to single- and multi-species cropping systems to facilitate characterization of the light environment. Different row layouts of an intercrop consisting of Bambara groundnut (*Vigna subterranea*) and proso millet (*Panicum miliaceum*) have been used as an example and the new opportunities presented by this approach have been analysed.

**Methods** Three-dimensional plant reconstruction, based on stereo cameras, combined with ray tracing was implemented to explore the light environment within the Bambara groundnut–proso millet intercropping system and associated monocrops. Gas exchange data were used to predict the total carbon gain of each component crop.

**Key Results** The shading influence of the tall proso millet on the shorter Bambara groundnut results in a reduction in total canopy light interception and carbon gain. However, the increased leaf area index (LAI) of proso millet, higher photosynthetic potential due to the C4 pathway and sub-optimal photosynthetic acclimation of Bambara groundnut to shade means that increasing the number of rows of millet will lead to greater light interception and carbon gain per unit ground area, despite Bambara groundnut intercepting more light per unit leaf area.

**Conclusions** Three-dimensional reconstruction combined with ray tracing provides a novel, accurate method of exploring the light environment within an intercrop that does not require difficult measurements of light interception and data-intensive manual reconstruction, especially for such systems with inherently high spatial possibilities. It provides new opportunities for calculating potential productivity within multi-species cropping systems, enables the quantification of dynamic physiological differences between crops grown as monoculture and those within intercrops, and enables the prediction of new productive combinations of previously untested crops.

## INTRODUCTION

Intercropping systems contain two or more species simultaneously and in close proximity for at least part of their growth season. The practice of intercropping is widespread in many areas of the world, including regions such as the tropics, where it can be the dominant form of agriculture (Kass, 1978; Beets, 1982; Francis, 1986; Vandermeer, 1989). Globally, most intercropping occurs on a small scale in resource-poor environments (Lithourgidis *et al.*, 2011), although adoption is increasing in developed countries such as the USA and areas of Europe (Jensen *et al.*, 2005; Blackshaw *et al.*, 2007; Hauggaard-Nielsen *et al.*, 2009). The production of a greater yield on a given piece of land (per equivalent component crop area) is the most commonly perceived advantage of intercropping systems (e.g. Willey, 1979, 1990; Vandermeer, 1989; Keating and Carberry, 1993; Dhima *et al.*, 2007; Mucheru-Muna *et al.*, 2010; Lithourgidis *et al.*, 2011). Often, growth resources such as light, water and nutrients can be more efficiently exploited within the intercrop system as a result of differences in the growth and competitive ability of the component crops (Midmore, 1993; Tsubo *et al.*, 2001). The benefits achieved will depend upon the crop combination used (for reviews on the benefits of intercropping see Malézieux *et al.*, 2009; Lithourgidis *et al.*, 2011; Brooker *et al.*, 2015), although cereal–legume intercroppin1g systems are commonly adopted as a synergistic system due to the nitrogen-fixing ability of the legume component, and provide increased yield under adverse conditions (Ofori and Stern, 1987; Dhima *et al.*, 2007).

Understanding and maximizing the productivity of intercropping systems is limited by the ability to accurately predict the resources captured and used by each of the components (Azam-Ali and Squire, 2002). One of the key features of an intercropping system is the complex canopy structure achieved within a multiple-species assemblage. Differences between the component crops in terms of developmental pattern and response to the competitive presence of other plants, planting density, row orientation and the local environment leads to differences in architectural features such as plant height, leaf size, shape and orientation plus the degree of foliage overlap (Keating and Carberry, 1993; Jaya *et al.*, 2001). Furthermore, canopy characteristics are not fixed, but will alter in response to the competitive presence of the other species (Keating and Carberry, 1993; Zhu *et al.*, 2016). This could be seen within a wheat–maize intercropping system, where key architectural features (including tiller production, tiller survival rate and leaf size) differed between sole-cropped wheat plants, wheat plants bordering maize plants (i.e. with maize one side and wheat the other) and wheat plants in the inner row (i.e. with wheat either side; Zhu *et al.*, 2016). This necessitates the development of methodologies that can incorporate this level of complexity and separate out responses of different component crops, or even different row responses.

**Fig. 1.**
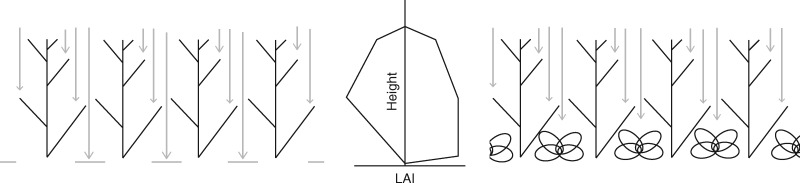
Theoretical example of light transmission through a monocropped canopy (left) versus an intercrop canopy (right). The estimated leaf area index (LAI) as a function of depth is given for each canopy.

The unique changes in architectural traits of intercropping systems also have consequences in terms of light transmission and absorption. Two or more species growing together in close proximity will intercept light both quantitatively and qualitatively differently from the equivalent monocrops (Vandermeer, 1989). As solar radiation provides the energy for photosynthetic processes, this will determine the potential for system productivity. Therefore, light interception and radiation use efficiency (biomass generated per unit radiation intercepted) provide two routes (either singly or in combination) of improving intercropping systems (Willey, 1990). Light interception can be improved both temporally and spatially by lengthening the period of soil coverage (i.e. extending the growing season; temporal complementarity) by one or more crop species, or by optimizing the distribution of leaf material within the canopy to maximize interception (spatial complementarity; Fig. 1) (Keating and Carberry, 1993; Brooker *et al.*, 2015). Separating spatial and temporal complementarity provides two benefits when considering and optimizing intercropping systems. Firstly, it highlights the importance of crop features that can lead to better resource use (e.g. plasticity; Zhu *et al.*, 2015, 2016). Secondly, it indicates two means by which resource use can be improved: greater resource capture and greater resource conversion efficiency (e.g. photosynthesis and transpiration). As well as increased light interception, rapidly growing crops that show early canopy closure could contribute to weed suppression (Midmore, 1993), a common problem in many cropping systems (Kutu and Asiwe, 2010). Earlier work on drought tolerance in Bambara groundnut cropping systems indicates that, early in the season, canopy cover is the major limitation to productivity, with reductions in leaf production and expansion negatively affecting dry matter production (Collinson *et al.*, 1999).

In the rest of this paper, we will focus on methods to optimize resource capture, namely light interception. However, in order to optimize systems further, accurate prediction of light interception within the system is first required. In theory, light capture by intercrops could be measured by similar methods to those used for sole crops (Azam-Ali and Squire, 2002). This could be through the use of photosynthetically active radiation (PAR) sensors, tube solarimeters, ceptometers and line sensors, placed such that they capture a representative sample of the crop system (Francis, 1986; Azam-Ali and Squire, 2002). Such methods could provide good estimates where the component crops are distinctly separate (i.e. the components occupy separate canopy volumes) and are relatively uniform, e.g. early in the growth stage or with sufficient distance between rows or in strip intercropping (McMurtrie and Wolf, 1983; Zhang *et al.*, 2014). However, they will be less accurate in more heterogeneous systems and will not able to capture small-scale features needed for high-resolution modelling. Traditional sensors can also be used for morphologically similar component crops (e.g. clover swards; Black, 1960, 1961), where it can be assumed that light interception can be attributed to the proportion of total leaf area of each component. Horizontal uniformity within canopies can be assumed in these instances (Duncan *et al.*, 1967) but, due to leaf clumping and row arrangement of crops, light penetration through the canopy is often underestimated. Where the different crops are structurally different, details of light interception by each component would be difficult to obtain and would require an extensive amount of sensors, and architectural differences between the component crops will lead to inaccurate predictions, as interception dependencies based on surface area will diverge for each component. Estimations in these cases will often result in large errors as a result of the spatial variation within intercrop canopies, particularly the row arrangements, orientations and distribution of foliage (Azam-Ali and Squire, 2002). Furthermore, heterogeneity is more common in low-resource agricultural systems, where intercropping is common. For these reasons, direct measurements of light interception by each component within a multi-species system are not economically or experimentally feasible (Sonohat *et al.*, 2002).

Contrary to direct measurement techniques, modelling approaches for estimating light within multi-species systems are advancing rapidly. To explore the relationships between intercrop design, canopy architecture and the resulting light environment and productivity, experimental results need to be combined with high-resolution methods of plant modelling (Zhu *et al.*, 2016). For morphologically distinct component crops, detailed measurements of the canopy structure of individual species can be combined with mathematical models of light patterning in order to model interception within intercrop canopies. Models in the literature range from low to high resolution, with low-resolution methods often assuming uniformity as discussed above. More accurate estimations of the light environment within an intercrop canopy require detailed, geometrically accurate three-dimensional (3D) models of component plants. Advances in computing power combined with affordability of both software and hardware has led to the development of a number of different techniques in order to capture plant structure (Watanabe *et al.*, 2005; Quan *et al.*, 2006; Song *et al.*, 2013; Pound *et al.*, 2014; Zhu *et al.*, 2015). One example of this is 3D reconstruction based on stereo cameras, which relies on digitizing a pre-existing structure, using a set of images as a basis (image-based reconstruction). Applications of image-based methods are diverse and include the estimation of canopy height, diameter and crown volume in isolated trees (e.g. Brown *et al.*, 2000; Phattaralerphong and Sinoquet, 2005; Patterson *et al.*, 2011) for the study of structural properties in sole cropping canopies (e.g. Ivanov *et al.*, 1995; Burgess *et al.*, 2015) or root systems (e.g. Lobet *et al.*, 2011) and for predictions of light interception or photosynthetic modelling (e.g. Andrieu *et al.*, 1995; Pound *et al.*, 2014; Burgess *et al.*, 2015). Accessible but high-resolution methods are increasingly needed to explore the complex temporal and spatial dynamics of light environment within canopies and have distinct advantages for multi-species intercrops, where spatial possibilities are greater.

In this paper we put modern methods for canopy reconstruction in the context of multi-species cropping systems and aim to test (1) whether image-based reconstruction can be used as a means to explore the light environment at high spatial resolution within a multi-species assemblage; (2) whether such methods provide new architectural and functional information (not achievable with previous manual measurements) when combined with ray tracing; and (3) whether suboptimal photosynthetic acclimation affects productivity of the systems. We have employed the reconstruction method of Pound *et al.* (2014), in which a 3D point cloud can be obtained with inexpensive SLR cameras and then automatically converted to a 2D leaf surface, for use in ray tracing (Song *et al.*, 2013). This method reconstructs the full canopy structure (not just the canopy surface) and ‘maps’ the complex patterns of light within the canopy over a whole day. We used examples from an intercropping system consisting of Bambara groundnut (*Vigna subterranea*) and proso millet (*Panicum miliaceum*) and their monocultures in order to assess light interception and potential productivity. The component crops were selected due to their compatibility as intercrops in terms of climate and soil requirements, differing growth durations and previous work carried out on legume–cereal systems, including pearl millet and groundnut (Willey, 1990). The tall (>1·2 m) proso millet combined with the much shorter (<50 cm), broadleaved Bambara groundnut crop provides an interesting combination for exploring the light environment due to shading effects, yet the shorter growth duration of proso millet (60–90 d compared with up to 150 d for Bambara groundnut) means that this shading would not be present for the whole growth season. This system therefore provides a means to explore the potential for both spatial and temporal complementarity. A modelling approach explores how different row layouts of the intercrop may influence the light environment and productivity in terms of total light interception and canopy carbon gain. This is the first such method to date that combines high-resolution modelling of ‘real’ intercrop canopy architecture (i.e. not simulated architecture) with a simulation of light to predict photosynthetic responses within the whole intercrop system.

## MATERIALS AND METHODS

### Plant material

Bambara groundnut X Dip C (*Vigna subterranea*) and proso millet (*Panicum miliaceum*; landrace from Sri Lanka) were sown directly into beds in the FutureCrop Glasshouse facilities, University of Nottingham Sutton Bonington Campus, UK, on 20 May 2014. This is an agronomy-style glasshouse designed and built by CambridgeHOK (Brough, UK) for the analysis of whole crop canopies under controlled conditions. It consisted of a concrete tank 5 m × 5 m × 1·25 m positioned at ground level. The tank was filled entirely with a sandy loam soil, extracted from local fields and sieved through a fine mesh. Plants were sown as four treatments: (1) sole Bambara groundnut, (2) sole proso millet, (3) 3 rows of Bambara groundnut to 1 row of proso millet (3:1) and (4) 2 rows of each species (2:2). There were 25 cm between rows, 25 cm between plants within rows of Bambara groundnut and 10 cm between plants within rows of proso millet. Irrigation was supplied using drip irrigation for 5 min, twice daily. Metal halide lamps provided additional lighting whenever the PAR fell below 200 μmol m^−^^2^ s^−1^ and a 12-h photoperiod (0700–1900 h) was maintained using blackout blinds. A constant temperature of 28 ±3 °C and relative humidity of 50–60  % was maintained throughout. As intercrops are generally grown under low-input agriculture, no additional fertilizer was supplied during the trial to either the intercrop treatments or the sole plots. The previous crop was rice. An image of the 2:2 intercrop treatment is given in Supplementary Data Fig. S1.

### Imaging and ray tracing

The 3D analysis and reconstruction of plants was done according to the protocol of Pound *et al.* (2014). Following photosynthesis measurements, the Bambara groundnut and proso millet plants (roots and shoots) were carefully removed from the glasshouse, placed into pots and taken to the imaging studio located nearby to prevent excessive movement and damage to leaves. For the light analysis, plants were removed 53 d after sowing (DAS) for imaging. Roots were supplied with water to prevent wilting. It was found that this process did not alter the key architectural or structural features of the plants. They were imaged within 1 h according to the protocol of Pound *et al.* (2014) and Burgess *et al.* (2015). An overview of the reconstruction process for an example Bambara groundnut and proso millet plant can be seen in Supplementary Data Fig. S2.

Three replicate plants representative of the morphology of Bambara groundnut and proso millet were taken and reconstructed to form the final canopies. The proso millet panicles were manually removed from the resulting mesh, as the reconstructing method is unable to accurately represent their form. Duplicating and randomly rotating the millet reconstructions in a 5 ×3 grid pattern, with 25 cm between rows and 10 cm between plants within rows, created the sole proso millet canopy. Sole Bambara groundnut canopies were similar but in a 3×3 grid pattern with 25 cm within and between rows. Intercropping canopies with different orientations (1:1, 2:1, 3:1, 4:1) were created similarly, with 25 cm between rows, 25 cm between plants within rows of Bambara groundnut and 10 cm between plants within rows of proso millet. An example of a full intercrop canopy reconstruction (3:1 row layout) is given in Supplementary Data Fig. S3. Reconstructed canopies consist of *n* triangles with coordinates of the *i*th triangle given by the vector $\left\{x_{i}^{1},y_{i}^{1},z_{i}^{1},x_{i}^{2},y_{i}^{2},z_{i}^{2},x_{i}^{3},y_{i}^{3},z_{i}^{3}|\}\right\}$, where coordinates *x* and *y* correspond to the coordinates on the ground and coordinate *z* corresponds to height above the ground.

Total light per unit leaf area for the *i*th triangle at time *t, L_i* (*t*), was predicted using a forward ray-tracing algorithm implemented in fastTracer (version 3; PICB, Shanghai, China; Song *et al.*, 2013). Latitude was set at 4·2, atmospheric transmittance at 0·5, light reflectance at 7·5 % and light transmittance at 7·5 %. The diurnal course of light intensities over a whole canopy was recorded at 6-min intervals. The ray-tracing boundaries were positioned so as to achieve further intercropping treatments (1:1, 2:1, 2:2, 3:1, 3:2, 4:1, 4:2). The software fires rays through a box with defined boundaries; when they exit one boundary (i.e. the side) they enter again from the opposite side, effectively replicating anything within the designated boundaries.

For a proof-of-concept canopy development time course, Bambara groundnut plants were grown in 5-L pots and proso millet in 3-L pots, which were sunk into the experimental plots; these were removed every 9 d (from 21 DAS) for imaging then replaced. This was due to space constraints in the glasshouse that meant that multiple plants could not be removed every 9 d. The same reconstruction process was carried out on these plants but they were not analysed for light interception (ray tracing).

### Physical and physiological measurements

#### Gas exchange

Measurements were made on glasshouse-grown proso millet and Bambara groundnut in plots in the same week in which the plants were imaged (early July 2014). Leaf gas exchange measurements were taken with an LI-6400XT infra-red gas-exchange analyser (LI-COR, NE, USA). The block temperature was maintained at 30 °C using a flow rate of 500 ml min^−^^1^. Light was provided by a combination of in-built red and blue LEDs. Light-response curves were taken on leaves that had not been dark-adapted. Illumination occurred over a series of nine PAR values between 0 and 2000 μmol m^−^^2^ s^−^^1^, with a minimum of 2 min and maximum of 3 min at each light level, starting at high intensities before reducing to zero. Light-response curves were taken at three different canopy heights, labelled top, middle and bottom for proso millet, and two different canopy heights labelled top and bottom for Bambara groundnut, approximately equidistant throughout canopy depth, with height above ground being noted. Three replicates were taken per treatment per crop (sole proso millet, sole Bambara groundnut, 2:2 and 3:1) for each canopy layer.

#### Ceptometer

**Fig. 2.**
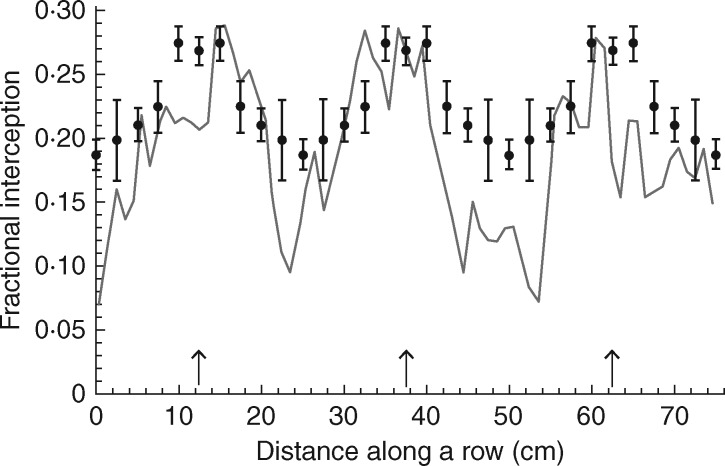
Validation of light interception in a sole Bambara groundnut canopy. Fractional interception was measured with a ceptometer (dots and bars, mean ± s.e.m.) and calculated from ray tracing (line) with distance along a row. Arrows indicate the location of the centre of the plants in a row.

To validate the light interception predicted by ray tracing, fractional interception was calculated at varying distances from the centre of a plant (i.e. along a row) using a ceptometer (AccuPAR) in a sole Bambara groundnut canopy. Light levels at the top and bottom of the plant canopies 0, 2·5, 5, 7·5, 10 and 12·5 cm from the centre of a Bambara groundnut plant were measured. Ten replicates were taken per location. This was compared with fractional interception calculated from ray tracing (Fig. 2).

### Statistics

Analysis of variance (ANOVA) was carried out on the fitted *P*_max_ parameter (maximum photosynthetic capacity) from light-response curves using GenStat for Windows, 16th edition (VSN International). Data were checked to see if they met the assumption of constant variance and normal distribution of residuals.

### Modelling

All modelling was carried out using Mathematica (Wolfram).

All triangles in each canopy reconstruction were assigned an identification code depending upon whether they were part of a proso millet reconstruction or Bambara groundnut. The ray-tracing files were then separated according to this identification code so the different component crops could be treated separately. A filter was applied to remove any data with photosynthetic photon flux density (PPFD) values below 0 (i.e. those outside the ray-tracing boundaries or in the simulated night time) and direct, diffused and scattered light was combined for each triangle and time point to give a single PFFD value.

Total canopy light interception per unit leaf area was calculated according to eqn (1):

(1) $${TL}_{LA}=\frac{\sum_{i=1}^{n}S_{i}\int_{5}^{22}L_{i}\left(t\right)dt}{\sum_{i=1}^{n}S_{i}}$$

where $S_{i}$ is the area of triangle *i.*

Total canopy light interception per unit ground area was calculated as light interception divided by the area of the ground each row of the component in the treatment took up (eqn 2):

(2) $${TL}_{LA}=\frac{\sum_{i=1}^{n}S_{i}\int_{5}^{22}L_{i}\left(t\right)dt}{N_{r}(\underset{i}{r.\max}x_{i}-r.\underset{i}{\min}x_{i})(\underset{i}{r.\max}y_{i}-\underset{i}{r.\min}y_{i})}$$

To predict the productivity of each of the intercrop treatments, as they would occur in the field, total canopy light interception per unit ground area for both components together was calculated as a ratio of the number of rows of each component together (eqn 3).

(3) $$TLI=\frac{N_{rows}^{BG}*{TL}_{LA}^{BG}+N_{rows}^{PM}*{TL}_{LA}^{PM}}{N_{rows}^{BG}+N_{rows}^{PM}}$$

where r refers to rows. For each depth (*d*, distance from the highest point of the canopy), we found all triangles with centres lying above *d* (eqn 4):
(4)
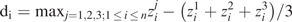


The response of photosynthesis to light irradiance, *L*, was calculated using a nonrectangular hyperbola given by eqn (5):

(5) $$F_{NRH}\left(L,\phi ,\theta ,P_{max},\alpha \right)=\frac{\phi \;L+\left(1+\alpha \right)P_{max}-\sqrt{{\left(\phi L+\left(1+\alpha \right)P_{max}\right)}^{2}-4\theta \phi L\left(1+\alpha \right)P_{max}}\;}{2\theta }-\alpha P_{max}$$

The non-rectangular hyperbola is defined by four parameters: quantum use efficiency, *ϕ*; convexity, *θ*; maximum photosynthetic capacity, *P*_max_; and rate of dark respiration, *R*_d_. We assumed that the rate of dark respiration is proportional to *P*_max_ according to the relationship *R*_d_ *= αP*_max_ (Givnish, 1988; Niinemets and Tenhunen, 1997; Retkute *et al.*, 2015). Curve fitting was carried out using the Mathematica command FindFit with a minimum constraint on *α* at 0·05 and *θ* at 0·6.

Carbon assimilation at triangle *i* was calculated by combining eqn (5) with the predicted PPFD at triangle *i* for each hour. Daily carbon assimilation, *P_i_* (eqn 6), was then calculated by integrating the rate of photosynthetic carbon uptake over the day and multiplying by the area of the triangle, *S_i_:*

(6) $$P_{i}=S_{i}\int_{5}^{22}F_{NRH}\left(L_{i}(t),\phi ,\theta ,P_{max},\alpha \right)dt$$

As each canopy was divided into three layers for proso millet and two layers for Bambara groundnut, each triangle from the digital plant reconstruction was assigned to a particular layer, *m*, according to the triangle centre (i.e. with triangle centre between the upper and lower limits of a layer depth). Carbon gain per unit leaf area, *C_l_*, was calculated as daily carbon assimilation over a whole canopy divided by the total surface area of the canopy according to eqn (7):

(7) $$C_{l}=\frac{\sum_{i=1}^{n}P_{i}}{\sum_{i=1}^{n}S_{i}}.$$

Carbon gain per unit ground area, *C_g_*, was calculated as daily carbon assimilation over a whole canopy divided by the area of the ground occupied by each row of the component in the treatment according to eqn (8):

(8) $$C_{g}=\frac{\sum_{i=1}^{n}P_{i}}{N_{r}\left(\underset{i}{r.\max}x_{i}-r.\underset{i}{\min}x_{i}\right)\left(\underset{i}{r.\max}y_{i}-\underset{i}{r.\min}y_{i}\right)}$$

where *r* refers to rows.

## RESULTS

### Validation of imaging and modelling

Previous studies validated the imaging and ray tracing techniques, showing that they are able to accurately and quantitatively predict physical properties within sole-cropped cereal canopies. The difference in leaf area using manual measurements and reconstructed plants has been shown to be low (4 % in Pound *et al.*, 2014, and 1 % in Burgess *et al.*, 2015) and similar percentages of leaf and stem material plus accurate leaf angles can be reproduced (Supplementary Tables S1 and S2 in Burgess *et al.*, 2015). Light interception throughout canopy depth has also been shown to be accurate (Fig. 5 in Burgess *et al.*, 2015). In this study we strengthen this: physical measurements were made to validate spatial differences in light interception. Fractional interception along a row in sole-cropped Bambara groundnut was calculated from ceptometer data and from modelled data; the results are given in Fig. 2. Good correspondence between measured and predicted values was seen. Despite this being a sole canopy, it has the same bimodal properties as seen in intercrops.

### The light environment

**Fig. 3.**
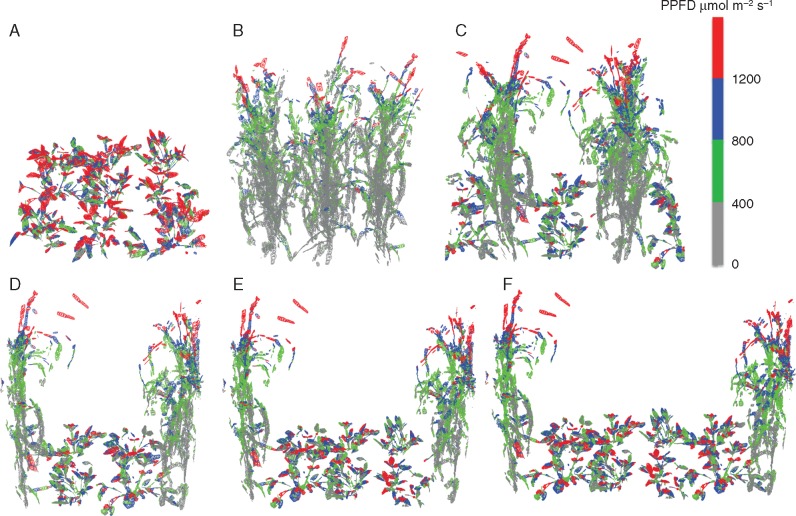
Representative reconstructed canopies with the maximum PPFD ranges colour coded for 1200 h. (A) Sole Bambara groundnut. (B) Sole proso millet. (C–F) Rows of Bambara groundnut:proso millet 1:1 (C), 2:1 (D), 3:1 (E) and 4:1 (F).

**Fig. 4.**
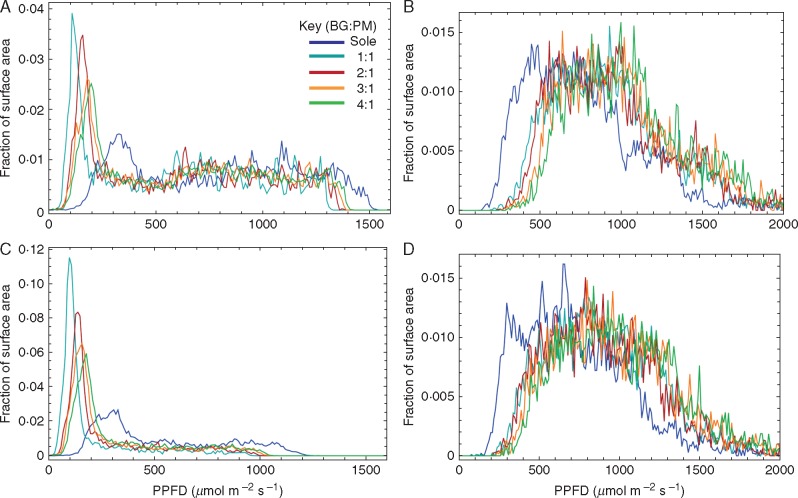
Frequency of PPFD values according to the fraction of surface area received at the top layer within each canopy. (A, C) Bambara groundnut; (B, D) proso millet. (A, B) 1200 h, direct light from above. (C, D) 1500 h, direct light from the side.

The light environment within the intercropping treatments is most easily visualized by colouring the leaf material in the reconstructions according to the light levels they experience (see Fig. 3 for values at 1200 h). As the reconstructed canopies are represented as a series of triangles, they may each be coloured according to the PPFD value from the ray-tracing output for any time of day. More red indicates high levels of light, whereas more grey indicates low levels of light. This is a useful way of instantly visualizing light distribution in different canopy arrangements across small spatial scales, which was not previously possible with techniques that integrate light over greater spatial scales. A quantitative method of visualizing the light dynamics between different treatments can be seen in Fig. 4. By plotting the average PPFD received as a function of the fraction of the surface area of each component canopy, we can see peaks in distribution indicating that large proportions of the canopy leaf area received similar levels of light. There was a shift in distribution towards a greater fraction of surface area under higher PPFD levels as the proportion of Bambara groundnut increased. This was due to the shading effects imposed by proso millet in the intercrop treatments. Contrary to this, there was a progressive shift in the opposite direction towards lower PPFD values for the sole proso millet relative to any of the intercropping canopies as less light was able to penetrate within and between the rows. This shows that increasing the ratio of Bambara to millet increases the amount of light received per plant for both species. The relationship between leaf area index (LAI) and total PPFD per unit leaf area along a row for the sole cropping and a 2:1 intercropping treatment is given in Supplementary Data Fig. S4; the position of the centre of plants in each row is given by arrows.

**Fig. 5.**
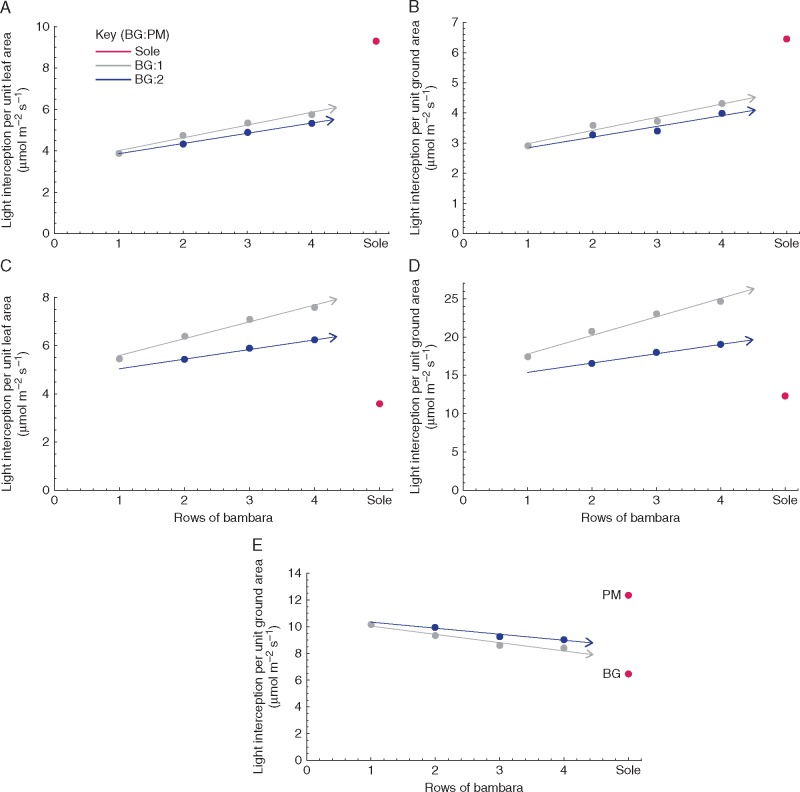
Modelled total canopy light interception over the course of the day for different intercrop treatments and corresponding sole crops (A, C) per unit leaf area and (B, D, E) per unit ground area. (A, B) Bambara groundnut. (C, D). Proso millet. (E) Both component crops. The number of rows of BG are shown along the *x* axis and the rows of PM are shown in the key as either 1 row (grey) or 2 rows (blue).

To quantify how much light each of the components and treatments received, total light interception was calculated (Fig. 5; eqns 1 and 2). On a unit leaf area basis, sole Bambara groundnut intercepted more light than sole proso millet; however, the opposite was seen on a per unit ground area basis, due to the much higher LAI of proso millet (LAI values are given in Table 1). Similar patterns could be seen when looking at each separate component on a per unit leaf area and ground area basis (Fig. 5A–D). For example, of the intercrop treatments tested within this study, both Bambara groundnut and proso millet exhibited greater light interception (per unit leaf area and unit ground area) in the 4:1 row orientation. As the number of rows of Bambara groundnut decreased, the total light interception also decreased. The greater number of rows of millet also reduced total light interception. However, to fully assess light interception by an intercrop, both components must be studied together (Fig. 5E; eqn 3). The average interception per unit ground area indicated that a sole proso millet canopy intercepted the most light and the sole Bambara groundnut canopy the least light of all treatments tested (monocrop and intercrop). Of the intercrop treatments, 1:1 gave the greatest light interception, with reducing interception with increasing number of rows of Bambara groundnut. These results are consistent with the LAI values for each of the treatments (Table 1), with the greatest LAI leading to the greatest total light interception value. Similarly to Barillot *et al.* (2011), we found a strong relationship between the component contribution to LAI and the PPFD intercepted (Supplementary Data Fig. S5). There was a tendency for higher PPFD interception by proso millet relative to contribution to LAI for all intercrop treatments.

**Table 1:** Total leaf area index (LAI) for each of the treatments. LAI was calculated as the area of all triangles [from both Bambara groundnut (BG) and proso millet (PM) reconstructions] within the ray-tracing boundaries divided by the ground area within the boundaries

Treatment	LAI
Sole BG	0·701258
Sole PM	3·42008
BG:PM	
1:1	1·97273
2:1	1·59127
2:2	1·90878
3:1	1·338
3:2	1·64266
4:1	1·25194
4:2	1·52017

### Assessing productivity

**Fig. 6.**
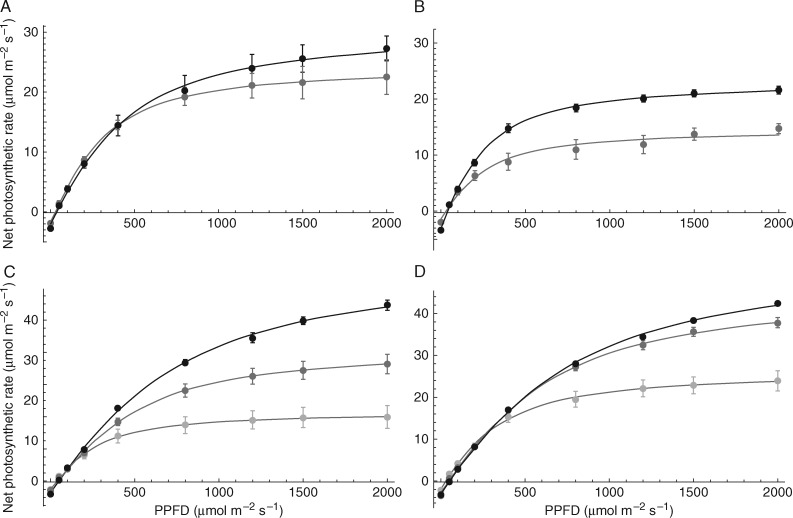
Example light response curves. (A, B) Bambara groundnut layers: top (black) and bottom (grey). (A) Sole plot and (B) intercrop (3:1) treatment. (C, D) Proso millet layers: top (black), middle (dark grey) and bottom (light grey). (C) Sole plot and (D) intercrop (3:1) treatment.

Intercepted light must be used efficiently, i.e. the proportion of light in excess of photosynthetic requirements should be as low as possible. The method described here is able to distinguish light distribution with high spatial resolution, and therefore photosynthesis modelling becomes highly accurate and presents more opportunities for calculating the proportion of excess light in different systems. Here we used an empirical model with light-response curves, measured at three different canopy layers for proso millet and two layers for Bambara groundnut. A non-rectangular hyperbola (eqn 5) was fitted to the experimental data in order to determine the quantum use efficiency (*ϕ*), convexity (*θ*) and maximum photosynthetic capacity (*P*_max_). Fitted curves are given in Fig. 6. These results are in broad agreement with previous studies on Bambara groundnut and C_4_ species (e.g. Dias-Filho, 2002; Cornelissen, 2005). The maximum photosynthetic capacity decreased with depth in the canopy for each of the component crops. Such responses are typical of canopy depth-dependent changes caused by light acclimation and leaf ageing (Murchie *et al.*, 2002). There was no significant difference in *P*_max_ for any layer between the intercrop treatments of sole cropping for either component crop.

The analyses in Figs 4 and 6 can be compared to see how the levels of photosynthesis matched light availability (see Supplementary Data Fig. S6 for overlaid graph). Generally speaking, the large peaks at low light levels in Fig. 6 will reduce canopy productivity since they match lower photosynthesis rates. The optimal position was at the point of light saturation of photosynthesis, which broadly for Bambara groundnut was between 600 and 800 μmol m^−^^2^ s^−^^1^ regardless of canopy position or cropping arrangement. However, Fig. S6 also shows the average canopy light level superimposed with light response curves for midday. Photosynthesis in most leaves was nearly saturated at mid-day in Bambara and position-ranked according to cropping pattern. The higher the proportion of Bambara in the system the more saturated was photosynthesis and the greater the potential proportion of excess absorbed light energy. In contrast, the proso millet crop was only part saturated even at 1000 μmol m^−2^ s^−1^, consistent with C_4_ physiology. The point at which saturation was reached was around the same value for all canopy positions. Greater spacing and light penetration (Fig. 4) resulted in a higher rate of light-saturated photosynthesis in lower canopy layers due to acclimation of photosynthesis (Fig. 6) (Anderson, 1995; Murchie and Horton, 1997; Murchie *et al.*, 2002). For Bambara groundnut the opposite was the case, with acclimation to low light reducing light-saturated photosynthesis in both cases. Therefore, the Bambara intercrop component would not be able to make use of higher direct light at midday. Additionally, comparison with Fig. 4 and the measured differences in light compensation point and dark respiration rates, which were small, suggests that they would not be substantially better at exploiting the lower light levels than the sole crop. Therefore, such suboptimal acclimation of photosynthesis in Bambara should play an important role in restricting productivity in intercrops.

**Fig. 7.**
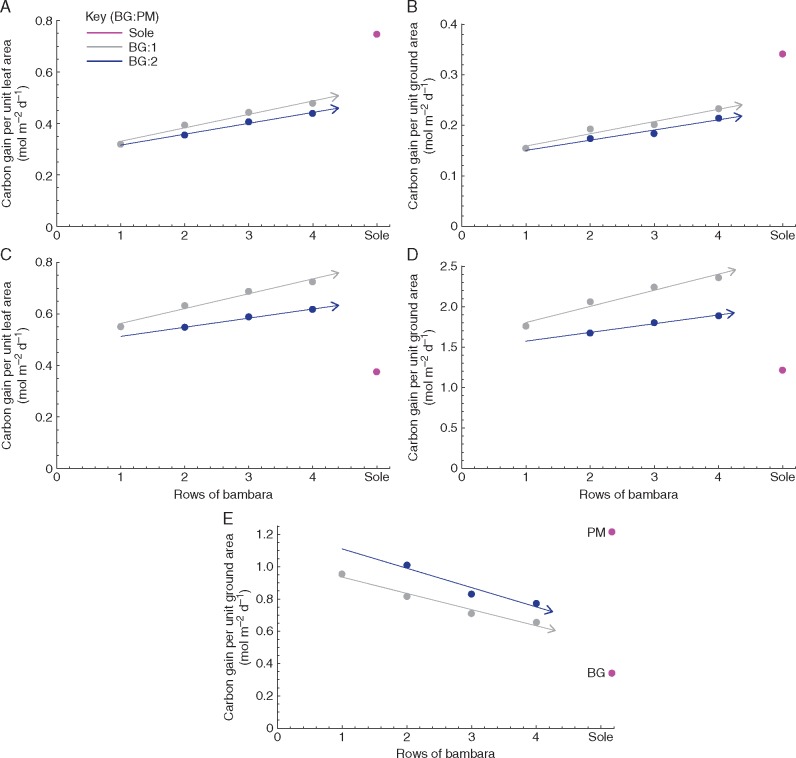
Modelled predicted carbon gain over the course of the day for different intercrop treatments and respected sole crops (A, C) per unit leaf area and (B, D, E) per unit ground area. (A, B) Bambara groundnut. (C, D) Proso millet. (E) Both component crops. The number of rows of BG are shown along the *x* axis and the rows of PM are shown in the key as either 1 row (grey) or 2 rows (blue).

To predict canopy productivity, daily net photosynthesis per unit leaf area and per unit ground area was also calculated for each component per treatment (eqns 6 and 7); results are given in Fig. 7. A line of best fit indicates the relationship between the number of rows of Bambara groundnut between each row of proso millet and the total canopy carbon gain for each component crop. The total canopy carbon gain per unit ground area (both components combined) was also calculated (eqn 8) and the results are given in Fig. 7E. Sole proso millet represents the maximal whole-canopy carbon gain per unit ground area available of all treatments, whilst sole Bambara groundnut represents the least productive, with intercropping values approaching this lower limit with increasing rows of Bambara groundnut. The declining carbon gain with increasing Bambara component showed a much steeper slope than that of intercepted light (compare Fig. 5E and 7E), indicating that the Bambara component was not able to compensate for the reduced millet component despite the increased photosynthetic productivity of the latter on a leaf area basis (Fig. 6). This is due to (1) the C_3_ pathway being relatively less productive than C_4_, and (2) acclimation to low light in the Bambara component when grown as an intercrop, such that it cannot exploit periods of high light.

## DISCUSSION

The structural complexity of intercropping systems containing contrasting plant types of different dimensions often results in a canopy with much greater spatial variation, which means that predicting system-level productivity is more difficult than for monocrop systems. This necessitates the need for new approaches to study intercropping systems that can capture this level of complexity and separate out responses of each component.

### High-resolution digital reconstruction as a method to explore the intercrop light environment

Here we describe a high-resolution method of capturing canopy geometry and exploring the light environment within an intercropping system. Without difficult and inaccurate manual measurements, we are able to (1) define structural and photosynthetic features throughout the vertical profile of the canopies; (2) separate each component of the intercrop by assigning identification codes to the reconstructions, and then combine them when required; (3) use different methods to visualize the shading influence of a tall component crop on a shorter crop; (4) accurately predict total light interception and include gas exchange data as a means to predict productivity within each of the systems; (5) acquire light data with high spatial and temporal resolution that can be used for dynamic photosynthesis measurements rather than integrated averages; and (6) make predictions for multiple different locations and treatments via modelling. This paper represents how simulations of different row patterning within an intercrop of proso millet and Bambara groundnut influence the light environment reaching each component crop and the resulting productivity.

Image analysis and reconstruction methods have previously been shown to accurately represent key physiological measurements and distinguish between different phenotypic traits, such as leaf curling, shape and area (e.g. Burgess *et al.*, 2015) and root morphology, geometry and topology (e.g. Lobet *et al.*, 2011). Image-based systems have practical and economic advantages due to the use of low-cost equipment; this means that digitizing canopies for 3D modelling *in silico* will become increasingly accessible. Furthermore, compared with other systems required for capturing plant structure (e.g. laser systems or phenotyping platforms), cameras are easily portable and can be used within the field. As image-based reconstruction works by digitizing existing plants, any structural differences found within the field-grown plants will be preserved in the final 3D model. The method could therefore be applied to study any structural differences and quantify differences in growth rate or development within the component crops as a result of intercropping. In this study, 53 DAS corresponded to an early vegetative stage of Bambara groundnut, and we did not witness any differences in structure between the intercrop and sole treatment plants.

*De novo* construction of 3D plants *in silico* would require knowledge of plant topology and multiple, intensive measurements of architectural features (i.e. leaf and stem length, leaf angle distributions etc.). Whilst few models are available for a select number of sole crops (e.g. Fournier *et al.*, 2003; Evers *et al.*, 2005; Valladares *et al.*, 2005; Song *et al.*, 2013), we are unaware of any such models specifically parameterized from intercropping data, although sole cropping models have been extrapolated for use in intercropping scenarios (e.g. Corre-Hellou, 2009; Barillot *et al.*, 2014). Furthermore, these rule-based methods can be time- and parameter-intensive (Fourcaud *et al.*, 2008; Vos *et al.*, 2010) and the averaged measurements can lead to large disparities from models containing explicitly described leaf angles (Sarlikioti *et al.*, 2011; for a review of functional structural plant modelling see Fourcaud *et al.*, 2008; Vos *et al.*, 2010; DeJong *et al.*, 2011). Rule-based reconstruction of 3D plants could also miss unique features of the canopy structure, which could determine light interception properties of the stand (Sonohat *et al.*, 2002). As canopy architecture is influenced by a number of different factors, including the competitive presence of other vegetation, features of a select crop grown within an intercrop setting are likely to differ from those of crops grown in monoculture; thus, existing models are unlikely to be suitable for application in such scenarios. It would be necessary to grow the plants in the intercrop setting to generate the correct morphology. This can be seen through differences in traits that confer plasticity on the plants and enable them to adapt to the situation in which they are grown (e.g. Reddy and Willey, 1981; Barillot *et al.*, 2011; Zhu *et al.*, 2015, 2016). Within a wheat–maize intercropping system, the yield advantage and increased land use efficiency (measured as the land equivalence ratio) of the intercropped system relative to sole wheat was attributed to the over-yielding of the border-row wheat (Zhu *et al.*, 2016). This over-yielding was a result of the plastic responses of the wheat to the intercropped environment; the plants exhibited higher tiller survival rate, a higher number of kernels per ear, higher N yield and larger sizes of leaves at the top of the canopy. This is consistent with the photosynthetic responses of millet seen in this study. An image-based approach would be able to capture the heterogeneity of component intercrops as it digitizes existing structures, and can achieve representative canopies over a much shorter time scale. This also means that plasticity present within the system adopted will also be reproduced in the final reconstruction.

We used an image-based reconstruction technique to study the partitioning of intercepted light between crop components in different planting arrangements in high spatial and temporal resolution. The proportion of light intercepted by each component varies according to LAI, its height and architecture. We show that any intercropping treatment that favours more rows of proso millet, or a taller component crop/component with higher leaf area, will have a greater total light interception, despite the shading influence and reduced interception by the Bambara groundnut component. The predicted light distribution given by ray tracing shows both spatial and temporal differences between each of our treatments. Achieving such high resolution, particularly with the ability to separate out responses of the intercrop components, would not be possible using manual measurements within the field/glasshouse and any attempts would require a large amount of sensors and data processing. For this reason, we were unable to validate the light simulation measurements for the intercropping scenario, but previous work has shown that the ray-tracing technique is able to accurately predict light interception within sole-cropped cereal canopies (Fig. 5 in Burgess *et al.*, 2015), and here we extend this to look at spatial differences along a row (Fig. 2).

Furthermore, we can make some novel predictions using photosynthesis measurements. A comparison between Figs 4 and 6 enables us to visualize how much light is in excess of photosynthesis requirements. Proso millet, being taller, becomes more productive due to absorption of light from all sides and exploitation of low solar elevations, while Bambara suffers from being shaded. Photosynthesis measurements reveal opposing patterns of photosynthetic acclimation in the two species. Acclimation is the process by which leaves adjust the composition and function of the photosynthetic apparatus (over a period of days) to enhance photosynthetic efficiency and productivity according to the prevailing light environment. Typically, low-light leaves have a lower light compensation point, lower photosynthetic capacity (*P*_max_) and lower dark respiration rate (Anderson, 1995; Murchie and Horton, 1997). Millet acclimates to the higher light intensities in the lower canopy positions (raises *P*_max_) and Bambara acclimates to the lower light in the intercrop (lower *P*_max_). This is likely to enable millet to be relatively more productive because Bambara will not be able to exploit high-light periods (1200 h) and does not demonstrate substantial changes in dark respiration or light compensation point, hence the advantage under low light is reduced. These photosynthetic data help to explain why the increased Bambara component was not able to compensate for the loss of proso millet despite the greatly increased photosynthetic capacity of the other per unit leaf area. It raises the intriguing possibility that superior ability to acclimate to shade is essential in a component intercrop and that we may need to select for varieties with such characteristics.

It is not sufficient to examine long-term changes such as acclimation alone; we need to understand photosynthesis as a dynamic process that responds locally and extremely rapidly to environmental fluctuations. Suboptimal responses on a time scale of seconds can affect canopy photosynthesis, e.g. via delayed relaxation of quantum yield of CO_2_ fixation (Zhu *et al.*, 2004). Traditional methods that integrate measurements of light and photosynthesis over spatial scales and long time periods render such physiological processes into an intractable black box. By studying 3D architecture in combination with ray tracing, we are able to accurately define the experimental framework within which photosynthetic dynamics operates, and this can include Rubisco activation, stomatal responses and photoprotection (Lawson and Blatt, 2014; Burgess *et al.*, 2015). A future system that measures 3D architecture and physiological status simultaneously would be paradigm shifting.

**Fig. 8.**
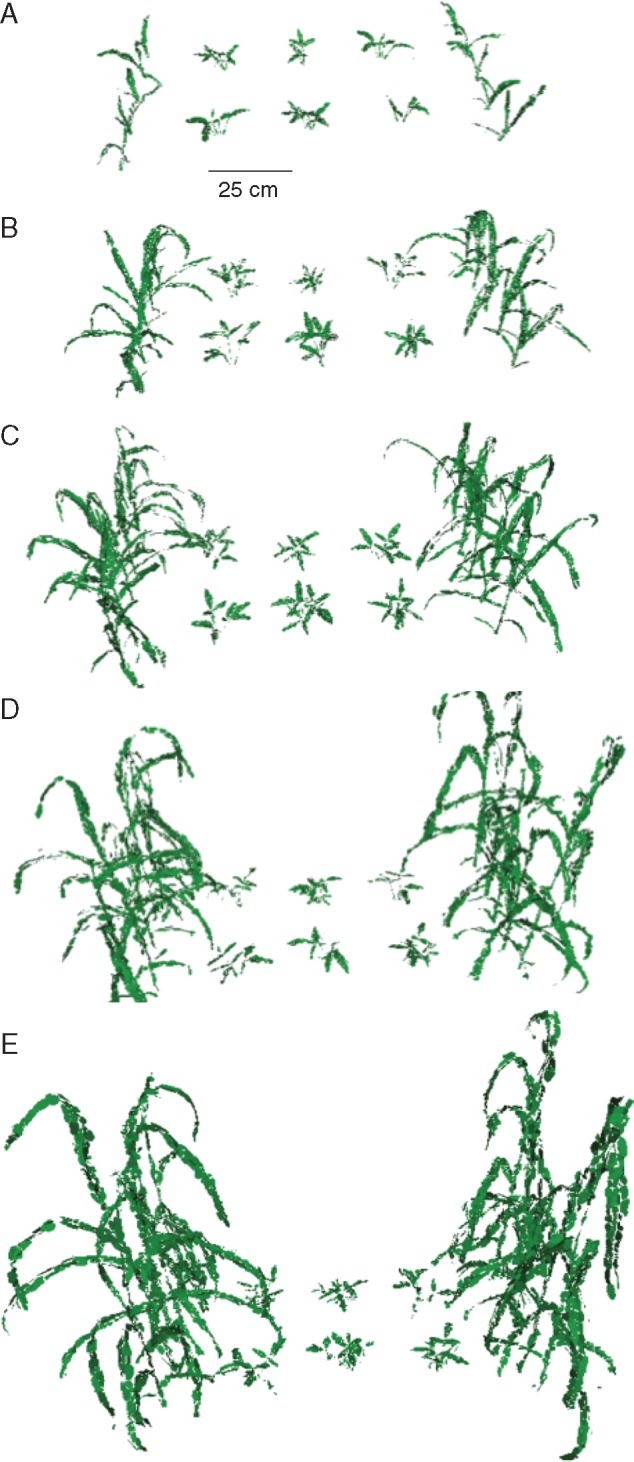
Reconstruction time course of a 3:1 (Bambara groundnut: proso millet) intercrop canopy development. (A) 21 DAS, (B) 30 DAS, (C) 39 DAS, (D) 48 DAS and (E) 57 DAS.

We have thus far considered a snapshot of a canopy in time. By capturing images at multiple times throughout the growth season, it is also possible to explore how the development and differential growth of each component may alter light patterning and productivity. Figure 8 shows the reconstructed canopy of a 3:1 intercrop every 9 d from 21 DAS. Time courses could be used in order to assess altered growth patterning as a result of the planting layout. This form of analysis could also be invaluable if it is known that one of the intercrop components (particularly the shorter component) has a specific light requirement at set stages during development, and thus planting date could be altered to fulfil these requirements. Alternatively, the plastic responses of a component crop to the competitive presence of another that differs in planting date could be explored.

### Studying light interception in heterogeneous canopies

The turbid medium approach to studying light attenuation through a canopy relies on two main assumptions: leaves are small and they are evenly dispersed throughout the canopy structure (Ross, 1981). However, homogeneity is rarely attainable in the field, either in sole cropping or multiple cropping systems, and departure from random leaf dispersion (i.e. through clumping) is common (for reviews see Myneni *et al.*, 1989; Baldocchi and Collineau, 1994; Valladares and Niinemets, 2007). Previous work on droughted Bambara groundnut (in a sole-cropped system) indicates how the non-uniformity of a canopy results in an inappropriate use of Beer’s law (Collinson *et al.*, 1999). The sparse canopy resulting from water stress, combined with changes in leaf orientation of individual plants, led to a non-random arrangement of leaves. This altered the light transmission towards a linear decay of light as opposed to exponential decay (Kasanga and Monsi, 1954). A study on the application of the turbid medium-based approach for the study of grass–legume intercropping systems indicated that the approach was suitable for certain situations; however, where there is considerable vertical heterogeneity in the canopy, more detailed canopy descriptions are required (Barillot *et al.*, 2011). Variability throughout the depth of the canopy results in differences in the vertical distribution of leaf area, with triangular distributions common in both sole and multiple cropping systems (Ross, 1981; Lantinga *et al.*, 1999; Sonohat *et al.*, 2002; Barillot *et al.*, 2011), although regular profiles can be seen for certain crops (e.g. Barillot *et al.*, 2011).

Studies on architectural characteristics within intercropping systems indicate how the assumption of homogeneity may not apply to a multiple cropping system even if the component crops are thought to be distinctly separate and the sole-cropped systems do exhibit regular dispersion (e.g. Sinoquet, 1993; Zhu *et al.*, 2016). Architectural traits such as leaf development and size, leaf angle distribution and tillering dynamics have been shown to be altered as a result of intercropping with maize (in a system containing six rows of wheat and two rows of maize) relative to sole cropping (Zhu *et al.*, 2016). Furthermore, differences were also seen within wheat that occupied the border rows of the intercrop (i.e. those immediately next to maize) relative to those that occupied the rows inside the wheat strip (i.e. those with wheat either side). The authors did not find any significant differences in the fraction of PAR penetrating to ground level at solar noon in the different canopy positions tested (apart from the position in the boundary between wheat and maize); however, it can be argued that the sampling approach adopted may not have been sensitive enough to locate any differences present. The authors did find significant differences in the PAR at ground level in the intercrop treatment relative to sole cropping. Furthermore, the pattern of change between the fraction of PAR at ground level over time differed between the intercrop and sole cropping treatments (Fig. 8 in Zhu *et al.*, 2016). Thus, within this strip cropping system, assuming independence would be inappropriate.

Because Beer’s law primarily describes the transmission of light through a canopy, in itself it is not enough to predict the light interception by individual components unless they are distinctly separate. For example, it will not be possible to infer the proportional interception by each crop component from light sensor data where the crops are overlapping in the same volume (Sonohat *et al.*, 2002). This can be manually overcome using the cumbersome visual point quadrat method (e.g. as applied to rye grass–clover mixtures in Lantinga *et al.*, 1999), but requires a large amount of data and processing. Alternatively, 3D models can be used to assess the light interception in a canopy setting. In particular, they is able to overcome the assumptions of random dispersion and requirement of small leaf size relative to plot size (Ross, 1981). Beer’s law and the visual point quadrat method account for the light attenuation through a canopy from a specific direction: directly above. However, in nature, the solar angle means that light predominantly enters from the side, and thus homogeneity is unlikely to apply in such situations. To manually measure light transmission accurately from all solar angles would require extensive data collection, and would only apply to the situation in which the data were collected.

In this study, distinct variations in leaf material distribution throughout both the horizontal and the vertical plane were present and their structural differences indicated patterns of light partitioning that could not be validated using manual measurements. These findings indicate the problems in assessing total light interception by a multi-species assemblage, or even within a highly heterogeneous monocropped canopy, and how existing techniques or ideas, such as Beer’s law, may not be appropriate.

### Designing the optimal intercropping system

Understanding the plant response to the environment in which it is grown, including the cropping system or practices adopted, will be critical in optimizing our agricultural systems. Traits that may confer optimal performance within one setting, e.g. in a monocrop, may be different from those that benefit another system, in this case an intercrop (Zhu *et al.*, 2015, 2016). One example can be seen with respect to leaf arrangements and traits that enable maximal light interception. Within monocropping systems, smaller, more erect leaves towards the top of the canopy and more horizontal leaves towards the bottom enable a greater distribution of light throughout all depths within the canopy (e.g. Duncan, 1971; Nobel *et al.*, 1993; Loss and Siddique, 1994; Peng *et al.*, 2008). This can be achieved in an intercrop by combining a tall erect canopy with a shorter horizontal canopy (Fig. 1) (Malézieux *et al.*, 2009). However, within an intercrop setting, direct light predominantly enters the canopy and reaches the shorter component from the side, as opposed to the top, thus negating the requirement for improved light transmission straight down. Within intercrop systems containing component crops of different heights, light transmission and interception must be balanced so as to enable transmission to the smaller component crop but still enable absorption by the taller component. The taller component will also be subject to higher light levels than in its monocropped counterpart, thus requiring other considerations, such as the prevention of damage caused by excess light (e.g. Burgess *et al.*, 2015). In Bambara groundnut, changes in leaf reflectivity and orientation to reduce incident radiation reaching the leaf surface are associated with drought tolerance, resulting in reduced transpiration and photoinhibition (Collinson *et al.*, 1999). However, if plants are less likely to incur damage from direct radiation as a result of their cropping system, these traits may not be required. This means that future breeding programmes may be required to take a more targeted approach to creating plant varieties for use in an intercrop system, and it is likely that these will diverge in traits required for monocropping systems (Zhu *et al.*, 2016).

Previous work on a Bambara groundnut–maize intercropping system at different planting densities highlights the importance of evaluating crop varieties for use within the intercropping system (Godwin and Moses, 2014). Whilst intercrop advantage [measured as land equivalence ratio (LER), land equivalence coefficient (LEC) and economic parameters; total variable costs (TVC), gross margins (GM) and net benefits] was found under all combinations tested, low yields of each component indicate the potential for further improvement of the system. The work shown here in terms of sub-optimal photosynthesis acclimation demonstrates this point. This improvement could be achieved through more optimal planting densities or through altered canopy architecture of the wheat component to reduce the dominance of the cereal. Thus, the ability to manipulate the light environment within a system will be critical in determining both the productivity of the final system and the balance between the component crops (Ofori and Stern, 1987; Godwin and Moses 2014; Keating and Carberry, 1993; Malézieux *et al.*, 2009).

Following accurate quantification of canopy architecture and the resulting light environment within a multi-species assemblage, a number of applications open up. Combining simulation data with small-scale trials (necessary to account for morphological adjustment of individual plants) aimed at collecting select measurements may provide the first stage in a process to help predict the optimal row layout of previously untested crop combinations. Whilst the simulations themselves would not be sufficient in accurately predicting the behaviour of the crops in the field, they may give an early indication as to which layout could prove the most productive in terms of light acquisition and potential carbon gain of the system. Using modelling approaches as a means to predict productivity enables both the assessment of extreme combinations of crops, but also enables different locations to be tested if climatic or weather data can be input. Such methods could provide an initial screening process for assessing intercrop combinations before more time-, labour- and space-incentive methods are used. Modelling of the same crops but under different abiotic limitations to their yield potential would also permit the synergistic effects of particular combinations to be identified and further investigated. Alternatively, coupling physical modelling with dynamic growth models could provide a means to link causative genomics with yield models, particularly where yield models are aimed primarily at optimizing sustainable yields in complex systems, such as intercrops.

There are other considerations when selecting an intercrop that may influence the crop combination chosen and the row layout, which may not coincide with the system that could achieve maximal light interception and productivity. Multiple cropping systems may provide a means to improve the outputs of an agricultural system that is limited by climate or environment, as is almost always the case for low-input agricultural systems, where intercropping tends to be practised. For example, relay intercropping (seeding a second crop into an existing crop before the harvest of the first crop) is able to extend the growing season and enable production of two crops in the same field, allowing producers to spread the production costs and fixed costs of equipment and land over two or more crops (Palmer *et al.*, 1993). The choice of component crops and their layouts may also be tailored depending on any environmental constraints of the land in which they are to be grown. Consumer habits and dietary requirements may also influence the quantities of crops required. Combining these other considerations into prediction models could achieve the best layout for both physiological and economic incentives of a set location.

### Concluding remarks

Three-dimensional reconstruction combined with ray tracing provides a novel, high-resolution method of exploring the light environment within an intercrop canopy and provides a platform for trying untested combinations and row layouts of multiple cropping systems. The contrasting component crops, in terms of both architecture and photosynthetic properties, would usually result in difficulties in predicting the productivity and light partitioning within such systems at high spatial and temporal resolution. However, using an image-based approach to plant reconstruction and the ability to separate out the different crop components when modelling means that quick, detailed assessments of the canopy light environment can be made. Hence, dynamic aspects of physiology can also be incorporated. This method, either alone or in combination with other data, provides an early platform for the assessment of new cropping systems.

## SUPPLEMENTARY DATA

Supplementary data are available online at www.aob.oxfordjournals.org and consist of the following. Figure S1: photograph of the 2:2 (Bambara groundnut:proso millet) intercrop treatment in the FutureCrop Glasshouse facilities, University of Nottingham, Sutton Bonington Campus, UK, prior to plant removal from imaging and reconstruction. Figure S2: example overview of the reconstruction process for (A) Bambara groundnut and (B) proso millet. The left-hand panel shows one of the original photographs of the plant (40+ used per plant), the middle panel shows the point cloud reconstruction derived from VisualSFM software (Furukawa and Ponce, 2010; Wu, 2011) and the right-hand panel shows the final reconstructed mesh derived from (Pound *et al.*, 2014). Figure S3: example of a full intercrop canopy reconstruction, 3:1 row layout. Three representative Bambara groundnut reconstructions and three representative proso millet reconstructions were duplicated and randomly rotated. Figure S4: relationship between leaf area index (LAI) and total photosynthetic photon flux density (PPFD) per unit leaf surface area along a row for (A) sole Bambara groundnut, (B) sole proso millet and (C) 2:1 (Bambara groundnut:proso millet) intercropping treatment. Figure S5: component contribution to LAI and total intercepted PPFD. Figure S6: frequency of light levels as a function of the fraction of the total surface area of the canopy received at 1200 h by the different treatments of (A) Bambara groundnut and (B) proso millet and the average irradiance, indicated by arrows, overlaid on the light-response curves of the sole (black) versus intercropped (grey) plants.

## Supplementary Material

Supplementary Data
