# Supplementary material for: Image-based 3D canopy reconstruction to determine potential productivity in complex multi-species crop systems
Source: Ann Bot. 2017 Jan 8;119(4):517–32. doi: 10.1093/aob/mcw242 (PMC5458713; doi:10.1093/aob/mcw242)
Supplement: Supplementary Data [file mcw242_Supp.zip › mcw242-suppl_data/aob-16567-s01.docx]

## Supplementary Figures

**Supplementary Figure S1:** Photograph of the 2:2 (Bambara groundnut: Proso millet) intercrop treatment in the FutureCrop Glasshouse facilities, University of Nottingham, Sutton Bonington Campus, UK, prior to plant removal from imaging and reconstruction.

**Supplementary Figure S2:** Example overview of the Reconstruction Process for (**A.**) Bambara groundnut and (**B.**) Proso millet. Left hand panel shows one of the original photographs of the plant (40+ used per plant), the middle panel shows the point cloud reconstruction derived from VisualSFM software (Furukawa & Ponce, 2010; Wu, 2011) and the right hand panel shows the final reconstructed mesh derived from (Pound *et al.*, 2014). N.B. The colourful circle in the two left panels is a calibration target used to optimise the reconstruction method and scale the final reconstructions back to the correct units.

**Supplementary Figure S3:** Example of a full Intercrop Canopy Reconstruction; 3:1 Row layout. 3 representative Bambara groundnut reconstructions and 3 representative Proso millet reconstructions were duplicated and randomly rotated. These were then arranged within the canopy with 25cm between rows, 25cm within the rows for Bambara groundnut and 10cm within the rows for Proso millet.

**Supplementary Figure S4:** The relationship between LAI and total PPFD per unit leaf surface area along a row for (A.) sole Bambara groundnut, (B.) sole Proso millet and (C.) 2:1 (BG:PM) intercropping treatment. Arrows indicate the location the centre of the plants in the row.

**Supplementary Figure S5:** Component contribution to leaf area index (LAI) and total intercepted photosynthetic photon flux density (PPFD). The relationship that represents an equal contribution by each component is given in the dashed line.

**Supplementary Figure S6:** Frequency of light levels as a function of the fraction of the total surface area of the canopy received at 1200 h by the different treatments of (A) Bambara groundnut (B) Proso millet and the average irradiance, indicated by arrows, overlaid on the light response curves of the sole (black) versus intercropped (grey) plants. This graph combines data presented in Fig. 4 and Fig. 6.
