# Supplementary figures and images for: Image-based 3D canopy reconstruction to determine potential productivity in complex multi-species crop systems
Source: Ann Bot. 2017 Jan 8;119(4):517–32. doi: 10.1093/aob/mcw242 (PMC5458713; doi:10.1093/aob/mcw242)

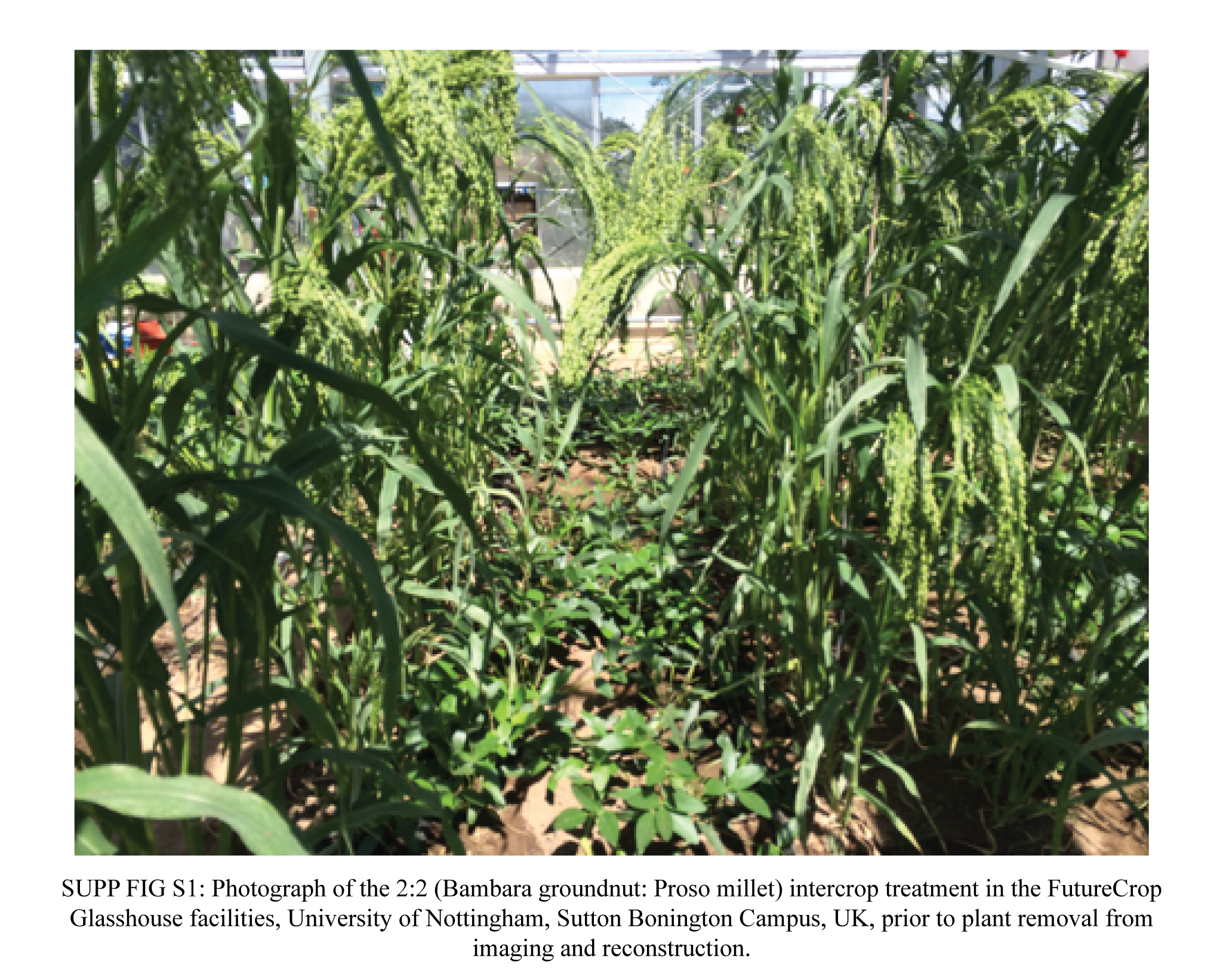

Supplement: Supplementary Data [file mcw242_Supp.zip › mcw242-suppl_data/aob-16567-s02.tif]

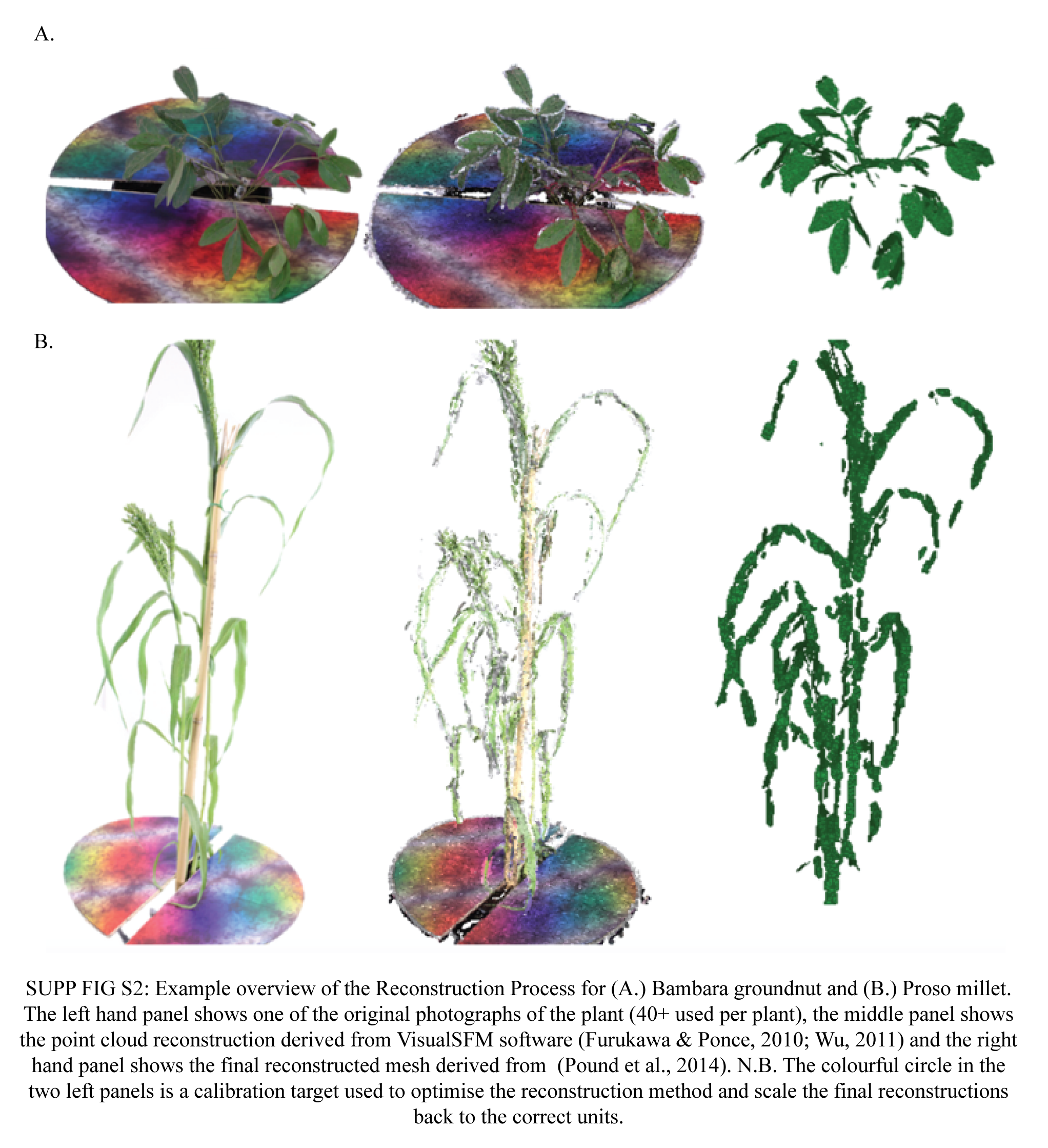

Supplement: Supplementary Data [file mcw242_Supp.zip › mcw242-suppl_data/aob-16567-s03.tif]

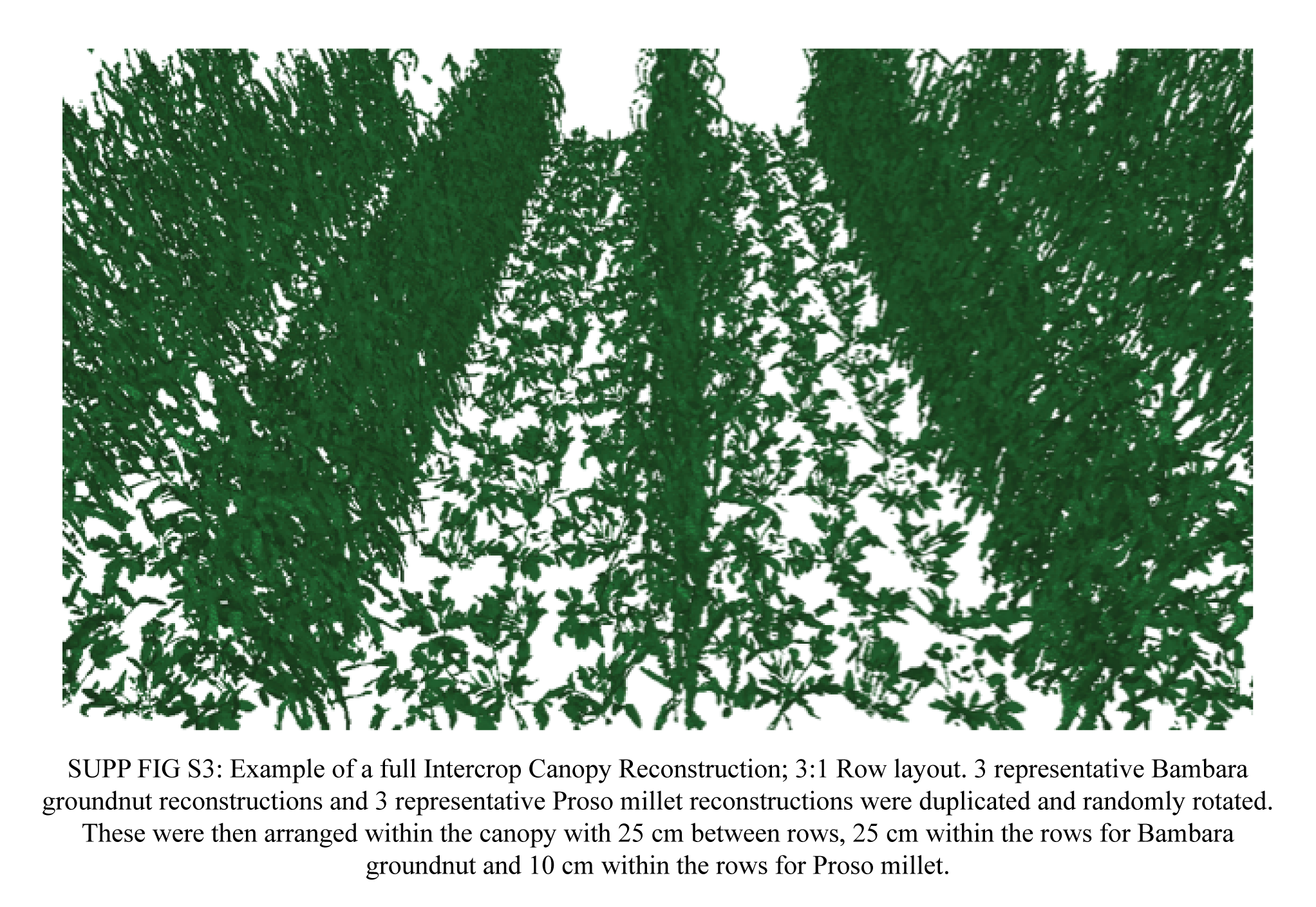

Supplement: Supplementary Data [file mcw242_Supp.zip › mcw242-suppl_data/aob-16567-s04.tif]

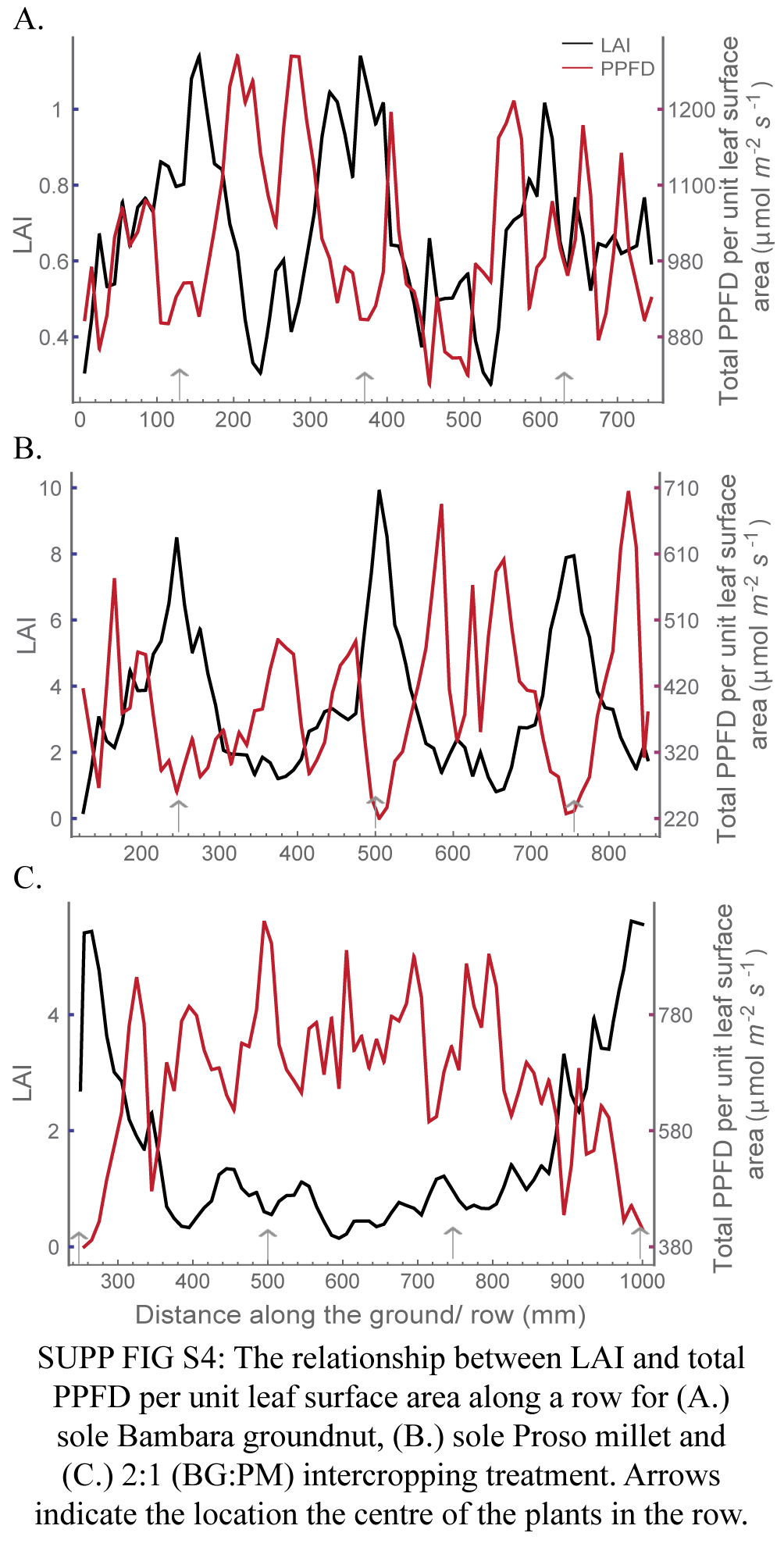

Supplement: Supplementary Data [file mcw242_Supp.zip › mcw242-suppl_data/aob-16567-s05.tif]

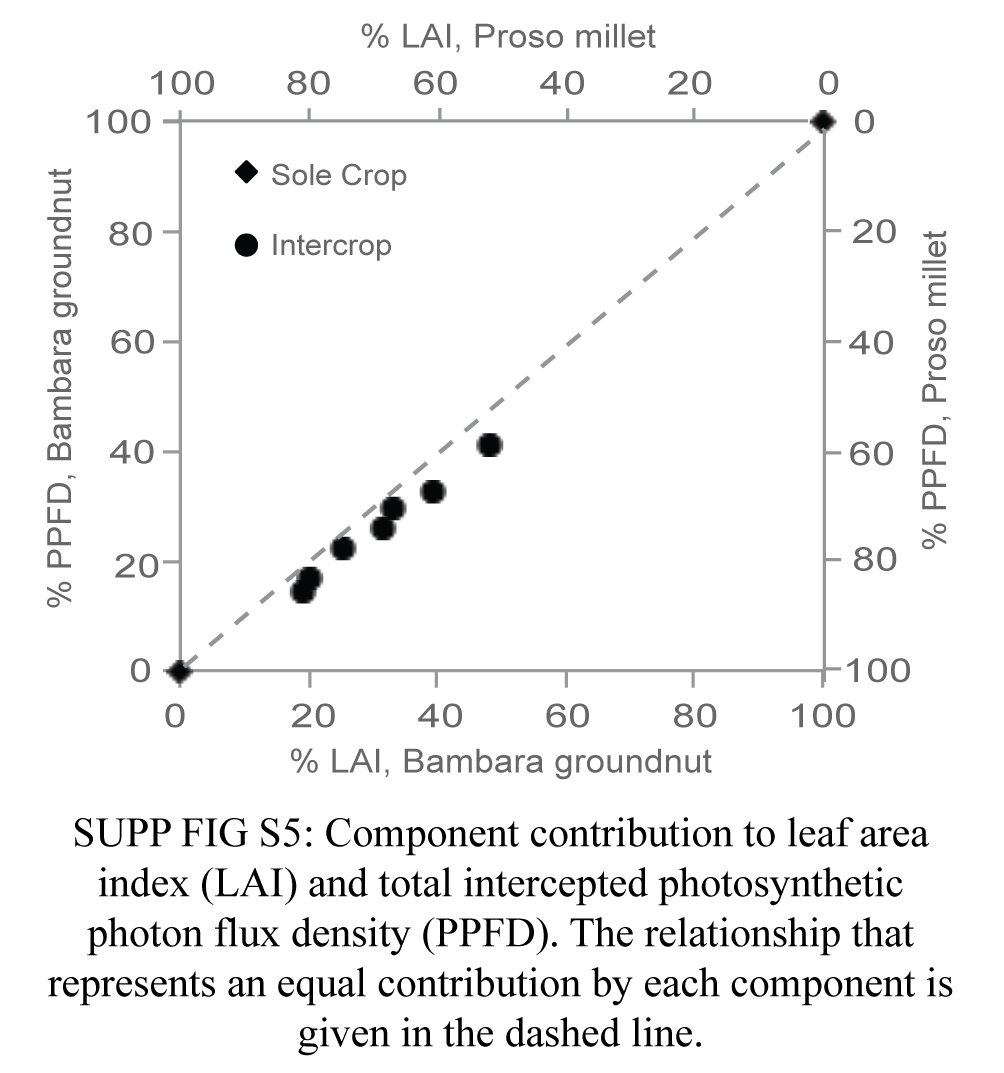

Supplement: Supplementary Data [file mcw242_Supp.zip › mcw242-suppl_data/aob-16567-s06.tif]

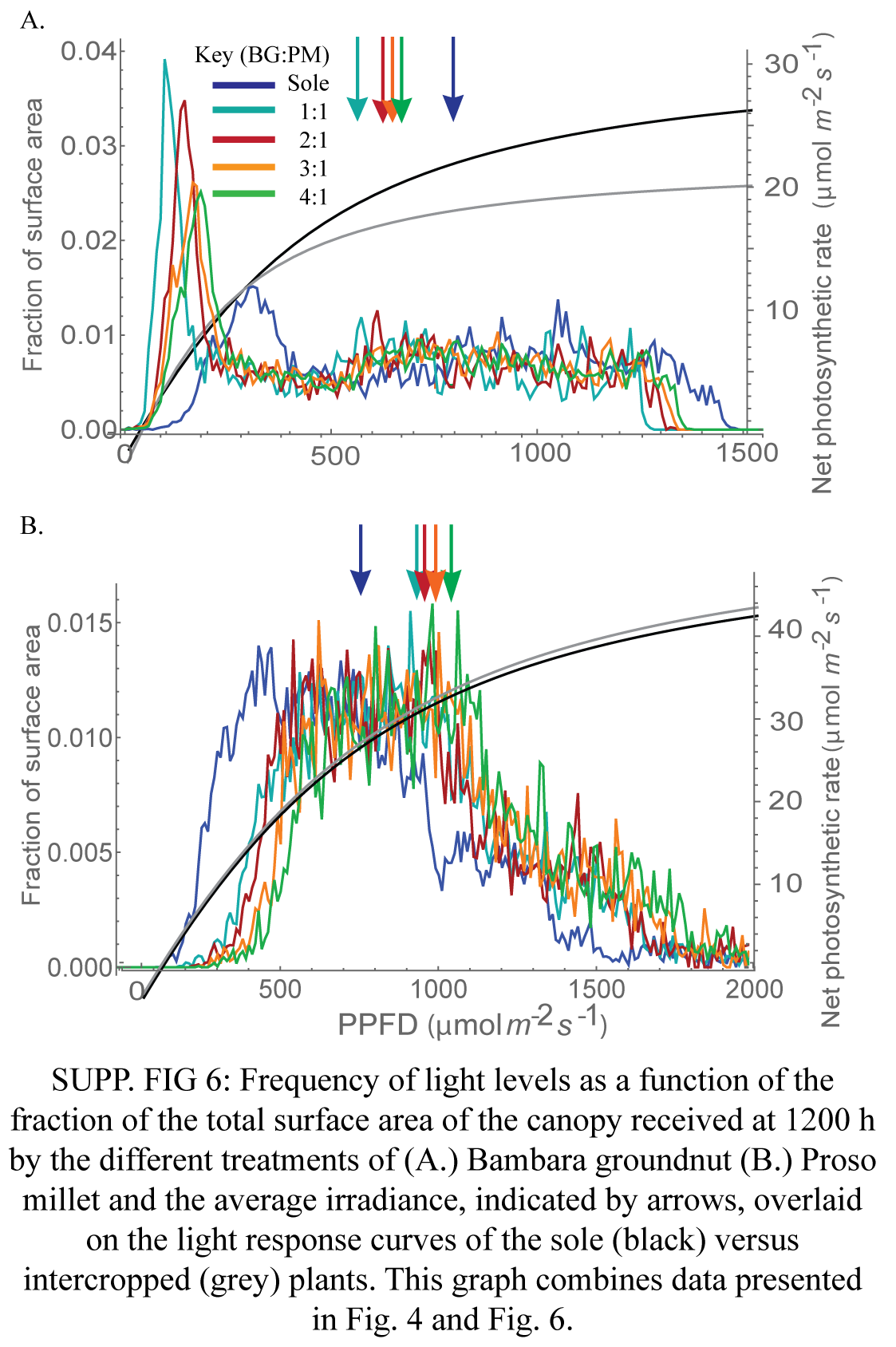

Supplement: Supplementary Data [file mcw242_Supp.zip › mcw242-suppl_data/aob-16567-s07.tif]
